# Changes in lower-limb neuromuscular performance from pre-season to the end of the early competitive period in elite male professional soccer players

**DOI:** 10.3389/fspor.2026.1722539

**Published:** 2026-03-05

**Authors:** Rodrigo Ferrari, William Borges da Silva, Leandro de Oliveira Carpes, Cíntia Lazzari, Anderson Donelli Silveira

**Affiliations:** 1 Postgraduate Program in Human Movement Sciences, School of Physical Education, Universidade Federal do Rio Grande do Sul, Porto Alegre, Brazil; 2 Sports and Exercise Training Study Group, Clinical Research Center, Hospital de Clínicas de Porto Alegre, Porto Alegre, Brazil; 3 Science, Health and Performance Department, Grêmio Foot-ball Porto Alegrense, Porto Alegre, Brazil; 4 Faculty of Physical Education, Universidade do Extremo Sul Catarinense, Criciúma, Brazil; 5 Cardiology Department, Hospital Moinhos de Vento, Porto Alegre, Brazil

**Keywords:** athletic performance, fatigue, football, sports training, vertical jump

## Abstract

**Background:**

The increasing compression of the pre-season period due to congested calendars may induce significant neuromuscular fatigue, potentially compromising player readiness for competition.

**Objectives:**

This study aimed to investigate the variation in neuromuscular performance in elite professional Brazilian male soccer players throughout the pre-season and at the end of the first competition of the year.

**Methods:**

Thirteen outfield soccer players (3 defenders, 5 midfielders and 5 forwards) from a single Brazilian first-division team were included in the study. Neuromuscular performance was evaluated using a comprehensive set of countermovement jump (CMJ) variables and isometric hip adduction/abduction strength at three time points: the start of the pre-season (T1), the end of the pre-season (T2), and following the end of first competition of the season (T3).

**Results:**

In T2, CMJ values significantly decreased compared to T1 for jump height, RSI modified, take-off peak force, peak power, and concentric peak velocity (*p* < 0.05). In T3, peak power and concentric peak velocity remained reduced, while eccentric duration increased compared to T1 (*p* < 0.05). Regarding hip strength, no significant changes were observed for adduction or abduction peak forces across the assessed time points (*p* > 0.05), indicating a maintenance of isometric strength levels.

**Conclusion:**

The findings indicate that an intensive and short pre-season induced a state of neuromuscular fatigue in elite soccer players, and the subsequent competitive period resulted in an incomplete recovery for most of the CMJ neuromuscular variables.

## 1 Introduction

Lower limb muscle power is essential for explosive actions such as jumps, sprints, accelerations, and changes in direction (1), making the countermovement jump (CMJ) an ideal tool for longitudinal monitoring in elite soccer due to its practicality and sensitivity (2, 3). Through variations in specific metrics such as jump height, modified RSI, and temporal phases, it is possible to infer the effects of training loads (4, 5). These variables provide objective data to support coaching decisions regarding training volume adjustments, tapering strategies, and recovery protocols, serving as a precise marker of readiness (2).

Athletes frequently exposed to intense training sessions and consecutive matches without adequate recovery periods are more prone to declines in their performance (4), increasing the risk of musculoskeletal injuries (6). The pre-season, a crucial preparation period preceding the competitive phase and aimed at developing athletes' physical fitness, has been increasingly shortened due to the sporting calendar with competitions in the first weeks of the season (7). This scenario compromises the execution of longer physical programs and the necessary recovery period before the start of the first competitions (8). Consequently, the insufficient tapering time may cause residual fatigue and incomplete adaptation, negatively impacting the athletes' neuromuscular performance.

Although some studies have described the longitudinal behavior of CMJ in soccer players (9), and although recent investigations have associated seasonal physical performance fluctuations with external workload metrics in elite soccer (10), the literature remains scarce regarding how specific neuromuscular strategies, particularly the concentric and eccentric CMJ phases and hip strength profiles, concurrently respond to the transition from a congested pre-season to the first competitive period. Given that these athletes already operate near their maximal performance capacity, their potential for further neuromuscular adaptations is relatively narrow. Nonetheless, the maintenance or enhancement of muscle power is essential for sustaining performance throughout the season. Therefore, this study aimed to analyze longitudinal fluctuations in CMJ and hip strength in elite professional soccer players during the transition from pre-season to the competitive phase. The hypothesis was that pre-season loads would induce neuromuscular fatigue, followed by a partial recovery during the competitive period.

## 2 Method

### 2.1 Study design and participants

This prospective longitudinal study with repeated measures monitored the athlete's standard routine. A non-probabilistic convenience sampling design was employed, encompassing all available athletes from a single professional club in the Brazilian first division who met the eligibility criteria. *A priori* sample size estimation was not conducted due to the applied nature of the study. The participants were full-time professional soccer players with a comprehensive training background extending from youth academies to the professional level, currently competing at the highest national level (Brazilian First Division). The cohort was classified as Tier 4 (Elite/International Level) based on their professional status, high training volume (6–10 sessions per week), and systematic participation in elite-level national and continental competitions. The inclusion criteria were: (a) being an outfield player (excluding goalkeepers) on the main squad; (b) participating in all three scheduled assessment sessions; and (c) being free from musculoskeletal injuries at the start of the data collection. A total of 35 athletes were initially recruited, 2 goalkeepers were excluded due to the specificity of their training loads; 10 players were excluded for joining the squad after the pre-season, and 10 were withdrawn during the follow-up period due to transfers (*n* = 4), national team call-ups (*n* = 2), or injuries (*n* = 4). The final sample consisted of 13 outfield players. While limited, this sample represents the complete eligible cohort available for longitudinal analysis, reflecting the practical limitations of elite professional settings. The detailed anthropometric and physiological characteristics of the participants are presented in Table 1. The study was conducted in accordance with the Declaration of Helsinki and approved by the local Institutional Review Board. All participants provided written informed consent before commencing any study-related procedures.

**Table 1:** Characteristics of elite male professional soccer players at the start of pre-season.

Variables	Soccer players (*n* = 13)
Age, years	27 ± 5
Anthropometric measures	
Body weight, kg	81 ± 6
Height, cm	180 ± 7
Body fat percentage %	10 ± 1
Cardiorespiratory fitness	
VO2_max_, mL kg^−1^ min^−1^	53 ± 3

Values are Means ± SD.

### 2.2 Experimental procedures

Baseline characterization included cardiopulmonary exercise tests (CPET) performed on a treadmill (Inbramed ATL, Porto Alegre, Brazil) using a ramp protocol and breath-by-breath gas analysis (Ultima CPX, Medical Graphics, St Paul, USA) (11). Body composition was assessed by ISAK-certified nutritionists using a digital skinfold caliper (CESCORF, Porto Alegre, Brazil). Four skinfold sites (anterior thigh, abdominal, triceps, and medial calf) were measured to estimate body fat percentage using the Reilly et al. (2009) equation (12, 13).

**Figure 1:**
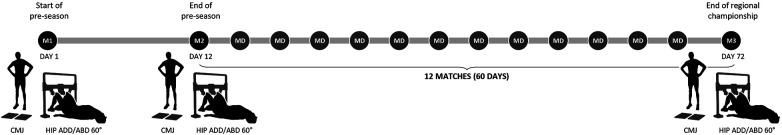
Experimental timeline comprising the 12-day pre-season (T1–T2) and the 60-day competitive period (T2–T3).

Outcomes were categorized into baseline characterization (assessed at T1) and longitudinal variables (assessed at T1, T2, and T3). Baseline measures included cardiorespiratory fitness and body composition, while longitudinal monitoring focused on CMJ and hip strength. The timeline comprised two periods (Figure 1). The pre-season phase (T1–T2 interval) spanned 12 days, consisting of 16 training sessions, one training match, and one day off. The mean daily exposure time was 78.5 min. The subsequent competitive period (T2–T3 interval) lasted 60 days, comprising 55 sessions (including 12 official matches) and five days off, with mean daily exposure decreasing to 50.9 min. The T3 assessment followed a 3-day complete rest period. The abbreviated pre-season was dictated by the club's competition calendar, a common reality for top-tier Brazilian teams facing limited transition periods and immediate competitive demands of regional tournaments in early January. The experimental protocol for this study was strictly designed to assess the neuromuscular response, and thus internal and external load control metrics (e.g., RPE, GPS-derived data) were not integrated into the current analysis.

Each testing session was preceded by a standardized 5-min warm-up consisting of joint mobility exercises and/or a low-intensity stationary cycling. The tests followed a fixed order: countermovement jump (CMJ) followed by a 5-min rest, then the isometric hip strength tests. To minimize the influence of circadian and environmental variables, all data were collected in the morning (between 08:00 and 10:00) in a climate-controlled environment. All assessments were conducted by a consistent team of three experienced professionals: two physical trainers and one physical therapist.

*Countermovement Jump* was assessed using a pair of portable force platforms (ForceDecks FDLite; Vald Performance, Australia). For the valid attempts, athletes started from an upright standing position with their hands fixed on their hips (akimbo position). On a verbal command, they performed a rapid countermovement to a self-selected depth to optimize individual stretch-shortening cycle mechanics, followed immediately by a maximal-effort vertical jump. Since these assessments are part of the club's routine, all players were previously familiarized with the protocol. Verbal motivation was provided during the tests. Participants performed three maximal attempts with a 30-s passive rest interval between them. The variables of interest were extracted by the system's software, and the maximum result (best attempt) of the three attempts was used for statistical analysis (14).

From the countermovement jump assessment, a comprehensive set of variables was extracted for analysis. These included: jump height (cm), determined using the impulse-momentum method; the modified reactive strength index (RSI-modified) (m/s) was calculated as the ratio of jump height to contraction time; temporal characteristics included the eccentric acceleration phase duration (s), which is the interval of the downward phase where the center of mass accelerates, and the eccentric Duration (ms), representing the total time of the downward movement; relative takeoff peak force (N/kg), defined as the maximum force generated during the concentric phase and normalized to body mass; vertical velocity at takeoff (m/s), the velocity at the moment of leaving the ground; power and work metrics consisted of relative peak power (W/kg), the maximum mechanical power generated normalized to body mass; several velocity variables were analyzed: concentric peak velocity (m/s), the maximum upward velocity; total work (J), the mechanical work performed during the concentric phase; and relative peak landing force (N/kg), representing the maximum ground reaction force absorbed during landing, also normalized to body mass (15).

#### 2.2.1 Isometric hip strength

Isometric adduction and abduction strength was measured using a groinbar-style strength testing system (Force Frame, Version 1.0; Vald Performance, Australia). Participants were positioned in a supine position with their knees flexed at 60° and hands crossed over their chest. The dynamometers were aligned with the femoral condyles. Prior to the maximal efforts, athletes performed a specific warm-up trial. Since this protocol is part of the club's routine, the athletes were already familiarized with the procedure. Participants then completed three 5-s maximal voluntary isometric contractions for each movement, which were performed in an alternating fashion: one adduction repetition was followed by 15 s of rest, and then one abduction repetition, repeating this sequence until three attempts for each movement were completed. Verbal motivation was provided during the contractions. For each movement (adduction and abduction), both the maximum (best attempt) and the average peak force of the three attempts were obtained for analysis (16). Previous research using this specific testing system has reported high intra-session reliability (Intraclass Correlation Coefficient >0.90) for hip strength measures in athletic populations (16).

From the isometric strength assessment using the Force Frame, two key variables were obtained. The first was Peak Adduction Force at 60° (N), representing the maximal isometric force produced during the bilateral hip adduction movement while the participant's hips were positioned at 60° of flexion. The second was Peak Abduction Force at 60° (N), which quantified the maximal isometric force produced during the bilateral hip abduction movement under the same positional condition. Absolute peak force values (N) were used for the analysis to monitor the direct force production capacity of the muscle groups.

### 2.3 Statistical analyses

Data distributions were analyzed using the Shapiro–Wilk test with analysis of histograms and Q–Q plots in combination. Data were expressed as means and standard errors for variables with normal distribution and 95% confidence intervals (95% CI). A one-way repeated measures Analysis of Variance (ANOVA) was used to investigate mean differences in primary study outcomes (e.g., CMJ and isometric hip strength metrics) across the fixed factor of time (e.g., pre-season baseline vs. end of pre-season vs. end of the regional championship). The assumption of sphericity was assessed using Mauchly's test, and the Greenhouse-Geisser correction was applied whenever this assumption was violated. To compare the main effects of time within each positional group, a two-way ANOVA was conducted for each condition (e.g., Defensor vs. Midfield vs. Forward) by time (e.g., pre-season baseline vs. end of pre-season vs. end of the regional championship). All *post-hoc* comparisons were adjusted using the Bonferroni correction.

Within-subject effect sizes were calculated using Cohen's *d* (mean difference divided by the standard deviation of the difference scores), and the interpretation of the effect size adopted was based on the following criteria: less than 0.50, small; 0.50–0.79, medium; and at least 0.80, large (17). Statistical significance was accepted at *p* less or equal than 0.050, and a trend toward significance was detected for *p* values ranging from 0.051 to 0.100. All analyses were performed using R (version 4.5.1; R Foundation for Statistical Computing, Vienna, Austria).

## 3 Results

Thirteen football players (age: 27 ± 5 years; body weight: 81 ± 6 kg; height: 180 ± 7 cm; body fat: 10 ± 1%; VO2_max_: 53 ± 3 mL kg^−1^ .min^−1^) participated in the study. Three athletes were defenders, 5 midfielders and 5 forwards. The mean daily exposure to training during the competitive period was reduced by 35% compared to the pre-season period.

**Figure 2:**
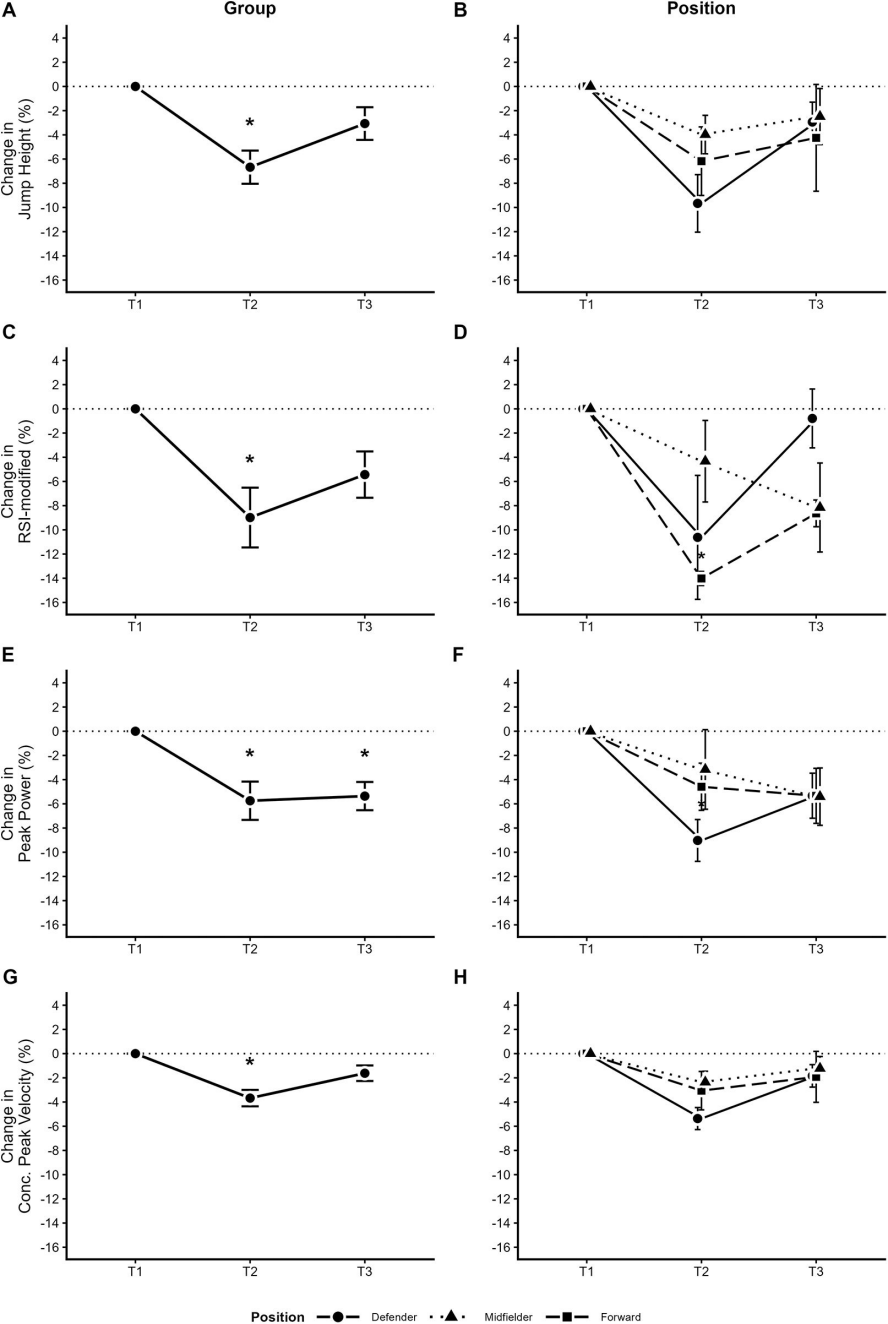
Longitudinal percentage changes in countermovement jump variables. The panels display the relative change from baseline across the three time points: start of pre-season (T1), end of pre-season (T2), and end of the regional championship (T3). Data are presented for the whole group (left panels: **A,C,E,G**) and by playing position (Right Panels: **B,D,F,H**). The specific variables are jump height **(A,B)**; RSI-modified **(C,D)**; peak power **(E,F)**; and concentric peak velocity **(G,H)**. Data are expressed as mean ± standard error. The legend identifies the specific playing positions (defenders, midfielders, forwards). *Indicates a significant difference from Pre-season baseline (T1) (*p* < 0.05).

The CMJ performance variables and the hip strength values throughout the different periods of the season are reported in Table 2. While Table 2 provides the complete descriptive statistics for all analyzed metrics, the text below highlights the statistically significant findings. For CMJ data, a significant main effect of time was found for jump height [*F*(1.85, 22.16) = 9.50, *p* = 0.001], RSI modified [*F*(1.91, 22.88) = 7.17, *p* = 0.004], eccentric duration [*F*(1.89, 22.74) = 4.37, *p* = 0.026], take-off peak force [*F*(1.66, 19.91) = 4.20, *p* = 0.036], vertical velocity at take-off [*F*(1.87, 22.49) = 9.97, *p* < 0.001], peak power [*F*(1.80, 21.63) = 10.70, *p* < 0.001], concentric peak velocity [*F*(1.92, 23.09) = 13.80, *p* < 0.001], and total work [*F*(1.95, 23.39) = 5.08, *p* = 0.015]. Conversely, no significant main effect of time was observed for eccentric acceleration phase duration [*F*(1.91, 22.96) = 2.69, *p* = 0.091] or peak landing force [*F*(1.73, 20.79) = 1.50, *p* = 0.246]. At the end of pre-season, values were significantly lower compared to the start of pre-season for jump height (*p* = 0.001), RSI modified (*p* = 0.004), take-off peak force (*p* = 0.036), vertical velocity at take-off (*p* = 0.001), peak power (*p* = 0.001), and concentric peak velocity (*p* < 0.001). At the end of the regional championship, peak power (*p* = 0.001) and concentric peak velocity (*p* < 0.001) remained lower compared to the start of pre-season, while eccentric duration showed a significant increase (*p* = 0.026). When comparing values from the end of the regional championship to the end of pre-season, a significant increase was found only for total work (*p* = 0.015). Position-specific analyses revealed no statistically significant differences among defenders, midfielders and forwards (*p* > 0.05) (Figure 2).

**Table 2:** Differences in countermovement jump parameters and hip adductor and abductor maximal strength of elite male professional soccer players across the three time periods.

Variables	Pre-season baseline	End of pre-season	End of the regional championship	*p-*value	Effect size
Countermovement jump					
Jump height Imp mom (cm)	42.2 ± 1.5	39.3 ± 1.3*	40.9 ± 1.6	**0.001**	1.2
RSI modified (ratio)	0.69 ± 0.03	0.62 ± 0.02*	0.65 ± 0.03	**0.004**	1.0
Eccentric acceleration phase duration (s)	0.30 ± 0.01	0.30 ± 0.01	0.32 ± 0.01	0.091	−0.6
Eccentric duration (ms)	447 ± 14	469 ± 15	487 ± 15*	**0.026**	−0.8
Take-off peak force (N/kg)	28.7 ± 1.1	26.9 ± 0.9*	27.9 ± 0.9	**0.036**	0.9
Vertical velocity at take-off (m/s)	2.87 ± 0.05	2.77 ± 0.04*	2.83 ± 0.05	**0.001**	1.2
Peak power (W/kg)	59.4 ± 1.8	55.8 ± 1.5*	56.2 ± 1.7*	**0.001**	1.2
Concentric peak velocity (m/s)	2.98 ± 0.05	2.87 ± 0.04*	2.93 ± 0.05	**<0.001**	1.4
Total work (J)	979 ± 48	952 ± 45	999 ± 42#	**0.015**	−0.8
Peak landing force (N/kg)	87.7 ± 9.4	80.2 ± 6.9	78.6 ± 7.4	0.246	0.5
Hip strength					
Adductor muscles					
Dominant leg	473.5 ± 13.6	433.8 ± 20.3	463.1 ± 20.8	0.116	0.6
Non-dominant leg	454.9 ± 13.2	429.0 ± 18.1	462.8 ± 19.3	0.173	0.4
Asymmetry (%)	4.3 ± 1.1	3.5 ± 0.7	4.9 ± 0.9	0.465	0.4
Abductor muscles					
Dominant leg	469.2 ± 14.1	458.2 ± 14.2	477.9 ± 13.5	0.241	0.4
Non-dominant leg	455.3 ± 12.8	449.4 ± 13.5	465.7 ± 15.1	0.381	0.3
Asymmetry (%)	4.2 ± 0.7	3.6 ± 0.5	6.4 ± 0.9^#^	**0.046**	0.8

Values are mean ± SE.

Imp mom: Impulse-Momentum method; Effect size values retain their sign to indicate directionality (positive values indicate a decrease from baseline; negative values indicate an increase from baseline).

Bold values indicates significant difference (*p* < 0.05).

* Indicates significant difference from pre-season baseline (*p* < 0.05).

^#^ Indicates significant difference from end of pre-season (*p* < 0.05).

Isometric strength values of hip adductors and abductors are presented in Table 2. For abductor muscle strength, a significant main effect of time was found for asymmetry (*p* = 0.046). *Post-hoc* tests with Bonferroni adjustment indicated no significant pairwise differences between time points. No other significant effects of time were observed for the remaining hip strength variables.

## 4 Discussion

This study investigated longitudinal changes in CMJ performance and hip adductor/abductor maximal strength in elite male professional soccer players across three key phases of the season: start and end of pre-season, and after the first regional championship. The main findings revealed significant fluctuations in some neuromuscular parameters, offering insights into the effects of training loads, fatigue, and competitive adaptation on performance.

The reduction in several performance-related variables at the end of pre-season suggests an acute accumulation of fatigue due to the high-intensity and high-volume training typically undertaken during this period (18). The magnitude of the observed decrements is functionally significant, as the large effect size for jump height (*d* = 1.2) translates to a loss of approximately 3 cm in vertical displacement, a change critical for success in aerial duels in elite competition. Elite athletes in the Italian football “Serie A” season had adaptations in athletic performance metrics which correlate with maintaining performance under competitive stress while avoiding excessive fatigue (19). The partial recovery of some CMJ parameters by the end of the regional championship may indicate that players adapted to the competitive demands, aligning with previous evidence that reduced training loads during the in-season, combined with the natural tapering effect around competition, potentially promote recovery and neuromuscular restoration (8, 20).

The CMJ assessment provides important variables that can aid in understanding neuromuscular fatigue. Barker et al. demonstrated that CMJ height is primarily associated with concentric phase output whereas reactive strength index (RSI) and jump time exhibit stronger relationships with unloading, eccentric, amortization, and concentric ground reaction force parameters (21). In the present investigation involving elite professional soccer players, a significant reduction in jump height, relative peak power, concentric velocities, and RSI was observed at the end of pre-season. By the end of the regional championship, jump height and RSI-modified values were restored to baseline levels; however, peak power and concentric peak velocity remained significantly reduced. Concurrently, eccentric duration increased gradually across the three time points, suggesting heightened demands on braking mechanics and a potential accumulation of neuromuscular fatigue. These results reinforce the notion that while concentric output metrics reflect changes in explosive capacity, eccentric and temporal variables, including eccentric duration and RSI, are more sensitive to neuromuscular fatigue and training load, thereby serving as valuable indicators of athlete readiness.

The sensitivity of jump height, RSI-modified, and eccentric duration to high training loads supports their inclusion in routine monitoring. Since these metrics showed significant alterations following pre-season, they serve as key indicators for adjusting training load. Given the congested Brazilian calendar, systematic assessment of these variables is crucial to monitor accumulated fatigue and guide recovery strategies. Spyrou et al. reported that professional players exhibited superior eccentric capabilities, characterized by deeper center of mass displacement, greater eccentric peak power, and higher eccentric peak velocity, when compared with semi-professional counterparts, showing that eccentric capabilities during vertical jump seem to differentiate professional and semi-professional players (3). In our study, eccentric duration increased significantly from the pre-season baseline to the post-championship period. This was accompanied by reductions in eccentric-related performance indicators, with only partial recovery, thereafter, showing that similar eccentric capabilities were impaired throughout the first trimester of the season. The need for longitudinal monitoring is also highlighted by Jarosz et al. (22), noting that power responses exhibit low reliability across microcycles. The lack of correlation between these changes and external match load assessed through GPS reinforces that individual responses are highly variable, necessitating the monitoring of multiple parameters.

Although the analyses do not reveal significant differences, it is possible to observe distinct behaviors between the positions throughout the season. As observed in Figure 2, seasonal fluctuations seem to affect more defenders and forwards than midfielders. Considering the small sample of this secondary analysis, future studies should better address this question using a larger sample of elite professional male soccer players as this potential difference can impact the training strategies for athletes of different positions.

The maximal strength of the hip adductors and abductors did not exhibit significant changes from the pre-season to the end of the first championship, but the abductor asymmetry fluctuation may be relevant since lower asymmetry is associated with reduced risk of groin-related injuries (23). Although the abductor strength remained relatively stable throughout the season, the higher variation in asymmetry during the competitive season may be dangerous as abductor weakness and imbalance may impact hip injuries (24, 25). The absence of fatigue in the adductor and abductor muscle strength can be explained by the lower contribution of these muscles compared to the muscles involved in the CMJ, especially in the main actions of the matches such as jumps and sprints.

This study is not without limitations. The relatively small sample size restricts the generalizability of our findings, and the inherent characteristics of professional football such as variability in training load, match schedules, and individual player availability may have influenced the outcomes. Another limitation was the absence of external load quantification via GPS technology. Although the specific running loads could not be integrated into the current analysis, limiting the direct dose-response assessment, the daily exposure time was rigorously recorded. This monitoring revealed a reduction in training volume during the competitive phase, providing a contextual basis for interpreting the variations in training load dynamics. Specifically, the limited cohort size constrained statistical power for subgroup analysis and renders the observed results highly specific to this single elite environment and seasonal structure. Nonetheless, the study has important strengths, including access to an elite sample of professional footballers, the use of highly sensitive equipment for neuromuscular performance assessments, and the adoption of rigorous methodological criteria for data collection and analysis. These aspects enhance the reliability of the results and provide valuable insights into performance fluctuations across different phases of the season. The combination of different neuromuscular assessments offers a comprehensive perspective on player monitoring, capturing acute fluctuations in neuromuscular performance of elite athletes.

In conclusion, the condensed pre-season induced neuromuscular fatigue evidenced by reduced CMJ metrics, and while jump height recovered during the competitive period, power and velocity outputs remained impaired, contrasting with the stability of hip strength throughout the period. These findings highlight the distinct response of explosive vs. isometric capabilities during the transition from a short pre-season to the first competitive period in elite players, enriching current knowledge by reinforcing the critical need to implement sustained weekly assessment of CMJ height and RSI-mod scores to identify fatigue patterns and guide immediate modifications to training intensity and recuperation methods in congested calendars.

## Data Availability

The raw data supporting the conclusions of this article will be made available by the authors, without undue reservation.
